# Antiseptic Surgery

**Published:** 1877-04

**Authors:** 


Antiseptic Surgery.—The operation of ovariotomy made by Dr. Marion Sims in November last was published in the *Reporter*. The tumor weighed fifteen pounds, and the operation lasted forty minutes. The carbolic spray was applied the whole time, the hands, instruments and sponges having been carbolized before commencing the extirpation.

In this publication it is claimed that Dr. Sims was the first to do this operation antiseptically; but the *London Lancet* denies the justice of his claim, and is "somewhat surprised that he should have entertained such a misconception."

Septicæmia, ulceration, and the formation of pus, which so frequently interfere with recovery from surgical operations, are prevented, it is said, by the antiseptic procedure. Conveniences for this purpose should, therefore, be at the command of surgeons for operations generally. While in all cases the process may not be required, yet, if the theory should be established as correct, the exceptional cases are not very certainly determined, and as no detriment is likely to result in any, its universal application would seem to be called for.

